# A Simulation Competition on Neonatal Resuscitation as a New Educational Tool for Pediatric Residents

**DOI:** 10.3390/children10101621

**Published:** 2023-09-28

**Authors:** Lorenzo Zanetto, Francesco Cavallin, Nicoletta Doglioni, Benedetta Bua, Sandro Savino, Giuseppe De Bernardo, Simone Pratesi, Paolo Ernesto Villani, Gary M. Weiner, Daniele Trevisanuto, on behalf of the Task Force on Neonatal Resuscitation of the Italian Society of Neonatology

**Affiliations:** 1 Department of Women’s and Children’s Health, University Hospital of Padua, Via Giustiniani, 3, 35128 Padua, Italy; lorenzo.zanetto@aopd.veneto.it (L.Z.); nicolettadoglioni@yahoo.it (N.D.); benedetta.bua89@gmail.com (B.B.); 2 Independent Statistician, 36020 Solagna, Italy; cescocava@libero.it; 3 Department of Medicine–DIMED, University of Padua, 35121 Padua, Italy; sandro.savino@unipd.it; 4 Department of Woman and Child, Ospedale Buon Consiglio Fatebenefratelli, 80122 Naples, Italy; pinodebtin@gmail.com; 5 Division of Neonatology, Careggi University Hospital of Florence, 50141 Florence, Italy; simone.pratesi@unifi.it; 6 Health Women Department, Poliambulanza Foundation, 25124 Brescia, Italy; paolo.villani@poliambulanza.it; 7 Department of Pediatrics-Neonatology, University of Michigan, Ann Arbor, MI 48109, USA; gweiner@med.umich.edu

**Keywords:** neonatology, resuscitation, simulation, competition, education, training

## Abstract

Background: Training programs on resuscitation have been developed using simulation-based learning to build skills, strengthen cognitive strategies, and improve team performance. This is especially important for residency programs where reduced working hours and high numbers of residents can reduce the educational opportunities during the residency, with lower exposure to practical procedures and prolonged length of training. Within this context, gamification has gained popularity in teaching and learning activities. This report describes the implementation of a competition format in the context of newborn resuscitation and participants’ perceptions of the educational experience. Methods: Thirty-one teams of three Italian pediatric residents participated in a 3-day simulation competition on neonatal resuscitation. The event included an introductory lecture, familiarization time, and competition time in a tournament-like structure using high-fidelity simulation stations. Each match was evaluated by experts in neonatal resuscitation and followed by a debriefing. The scenarios and debriefings of simulation station #1 were live broadcasted in the central auditorium where teams not currently competing could observe. At the end of the event, participants received an online survey regarding their perceptions of the educational experience. Results: 81/93 (87%) participants completed the survey. Training before the event mostly included reviewing protocols and textbooks. Low-fidelity manikins were the most available simulation tools at the residency programs. Overall, the participants were satisfied with the event and appreciated the live broadcast of scenarios and debriefings in the auditorium. Most participants felt that the event improved their knowledge and self-confidence and stimulated them to be more involved in high-fidelity simulations. Suggested areas of improvement included more time for familiarization and improved communication between judges and participants during the debriefing. Conclusions: Participants appreciated the simulation competition. They self-perceived the educational impact of the event and felt that it improved their knowledge and self-confidence. Our findings suggest areas of improvements for further editions and may serve as an educational model for other institutions.

## 1. Introduction

Nearly 10% of all newborns require some form of resuscitation at birth, with less than 1% needing chest compression or emergency medications [1,2]. The quality of care provided at the time of transition from fetal to neonatal life can have a meaningful impact on the outcome for these newborns. A successful resuscitation requires knowledge, procedural skills, and non-technical skills such as effective teamwork and confidence [1].

To achieve proficiency in resuscitation, educational programs have been developed for health care providers, using simulation-based learning to build skills, strengthen cognitive strategies, and improve team performance [3,4,5,6]. This has been shown to be utterly important for residency programs where reduced working hours and a high number of residents can reduce the educational opportunities during the residency, with lower exposure to practical procedures and prolonged length of training [7,8]. Recently, simulation-based medical training has profited from technology advancements, with virtual reality and high-fidelity simulation offering the participant the opportunity of a physical immersive environment and interactive experience [9].

Gamification has gained popularity in teaching and learning activities [10], as this process boosts participants’ engagement by integrating aspects of game design and competition [11,12,13]. Literature offers encouraging examples of onstage competitions (named “SimWars”) where two teams face the same scenario and a referee panel or audience declares the winner that moves to the next round [11,14,15,16]. For example, Ingrassia et al. organized two editions of a simulation competition on emergency medicine in 2015 and 2016 involving Italian residents from any residency program [11]. The participants found the event a valuable learning experience that improved their self-confidence in managing emergency scenarios. 

Since competitions on neonatal resuscitation have not been reported yet, we planned a simulation competition on neonatal resuscitation for Italian pediatric residents, which took place at the University of Hospital Padova (Italy) in February 2023. In this report, we describe the implementation of this competition format using high-fidelity simulation in the context of newborn resuscitation and participants’ perceptions of the educational experience.

## 2. Methods

The manuscript was prepared following the reporting guidelines for health care simulation research [17]. The simulation competition (called “Neonatal Resuscitation Competition”) took place from 16–18 February 2023 at the University Hospital of Padova (Padova, Italy) and was conceived by the Task Force on Neonatal Resuscitation of the Italian Society of Neonatology. In October 2022, all 35 Italian Pediatric residency programs were invited to participate by enrolling a team of three residents of any post-graduate year. The team members were chosen by the local residency trainers among the residents who volunteered. The event organizers did not set a minimum amount of time and training in the neonatal intensive care unit or the delivery ward prior to the competition as an inclusion criterium, but any decision on this aspect was left to the local residency trainers when recruiting the team. The 8th edition of the textbook of neonatal resuscitation [1] was suggested as the reference text for training before the event. The Ethics Committee of the University Hospital of Padova (Italy) deemed that a formal ethical approval was not required for the publication of the post-event survey, which was approved by the medical director of the University Hospital of Padova (Italy). Written informed consent was obtained from the participants.

### 2.1. Competition Design

The competition was planned as a 3-day event including an introductory lecture, familiarization time with the event equipment and competition phase. The introductory lecture was scheduled the morning of the first day and focused on “tips and tricks” regarding neonatal resuscitation by an expert neonatologist. After the lecture and the official presentation of the teams, the participants had the opportunity to familiarize themselves with the high-fidelity manikins, the infant warmers and the rest of the equipment that would be used during the matches. The competition phase was designed as a tournament-like event with five high-fidelity simulation stations where the teams competed in five rounds (qualification round, round of 16, quarterfinals, semifinals, and finals). The first three teams received a trophy (a wooden logo of the event carved by a local artist). The scenarios and debriefings of simulation station #1 were live broadcasted in the central auditorium where teams not currently competing could observe. This remote observation area was intended to provide an additional opportunity for participants to learn from the other teams’ experiences and benefit from hearing their debriefings. The judges were the members of the above-mentioned Task Force on Neonatal Resuscitation of the Italian Society of Neonatology, with Prof. Gary Weiner as the external expert. The teams were divided into five preliminary groups (one for each simulation station). In the first round, each team of three residents competed against the other teams in their group in a single round-robin format. The winner and runner-up of each group advanced to the 16-stage round. Each simulation competition included a randomly assigned case scenario of newborn resuscitation and had a maximum duration of 10 min. At the end of each simulation, a 20 min debriefing was carried out by two judges, and the team with a higher rank on the evaluation form (Supplementary File S1) won the match. Both teams competing in the same match received the same scenario and were debriefed simultaneously. Throughout the competition, a technical skill fair was available for the residents to practice their manual skills, including face mask ventilation, laryngeal mask insertion, and endotracheal intubation with coaching from local instructors. The list of case scenarios is summarized in Table 1. The 8th edition of the American Heart Association and American Academy of Pediatrics Textbook of Neonatal Resuscitation [1] was used as a reference text for the development of the assessment tool and for creating the clinical scenarios.

### 2.2. Performance Assessment

Two judges independently assessed the performances of each team using an evaluation form (Supplementary File S1) that was prepared before the event by the task force. For each item on the form, one point was assigned if the team correctly performed the skill, otherwise no points were assigned. Both technical and non-technical skills were evaluated. The judges were assigned taking into account the provenance of the competitors and they did not evaluate a match involving teams from their university/hospital. 

### 2.3. Resuscitation Stations

The stations were placed in different rooms. Each station included a high-fidelity manikin of a term neonate (Laerdal Newborn Anne^®^, Laerdal, Stavanger, Norway or Gaumard Super Tory^®^, Gaumard Scientific Company Inc., Miami, FL, USA) and an infant warmer (Alhena Infant Warmer, Ginevri srl, Albano Laziale (Roma), Italy or Resuscitaire, Draeger, Drägerwerk AG & Co., Lübeck, Germany). In each room, the participants had access to complete monitoring and intervention devices for newborn resuscitation as described in the reference textbook’s standard equipment list.

### 2.4. Participants’ Perceptions of the Experience

Each participant received an online survey at the end of the event (https://forms.gle/DeZRpewpSaB9pQzP8, accessed on 30 May 2023). The survey collected information on the team and logistics, training before the competition, satisfaction about the event, self-perceived confidence, and take-home messages (Supplementary File S2). 

### 2.5. Data Analysis

Data were summarized as the frequency and percentage (categorical data) or median and interquartile range (numerical data). Participants’ satisfaction, self-confidence, and opinion on take-home messages were measured using Likert scales. A comparison of self-confidence between the first and last match was performed using the Wilcoxon test, and a *p*-value less than 0.05 was considered statistically significant. Data analysis was performed using R 4.3 (R Foundation for Statistical Computing, Vienna, Austria) [18]. 

## 3. Results

### 3.1. Participants 

Overall, 31/35 (89%) Italian residency programs in Pediatrics participated in the event, and 81/93 (87%) participants completed the online form on their perceptions of the educational experience. They were pediatric residents in the second (*n* = 2, 2%), third (*n* = 19, 24%), fourth (*n* = 27, 33%) or fifth (*n* = 33, 41%) post-graduate year. Thirty-one (38%) played only in the qualification round, while 28 (35%) qualified for the round of 16, nine (11%) for the quarterfinals, and 13 (16%) for the semifinals/finals.

### 3.2. Logistics

To participate in the event, 65 participants (81%) were excused from scheduled hospital shifts, 10 (12%) used educational leave days, and five used their personal vacation days (6%) (one participant did not respond). For 78 participants travelling from another city, their university reimbursed travel costs (*n* = 32, 41%), meal costs (*n* = 23, 29%), and hotel costs (*n* = 33, 42%).

### 3.3. Training

**Figure 1:**
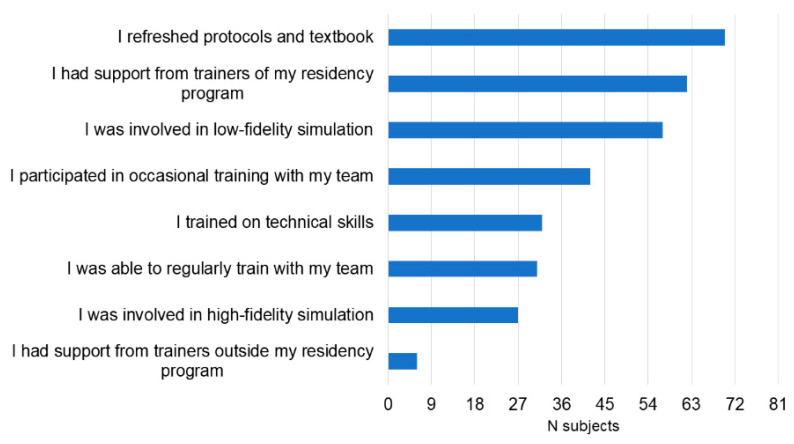
How the participants trained before the event.

**Figure 2:**
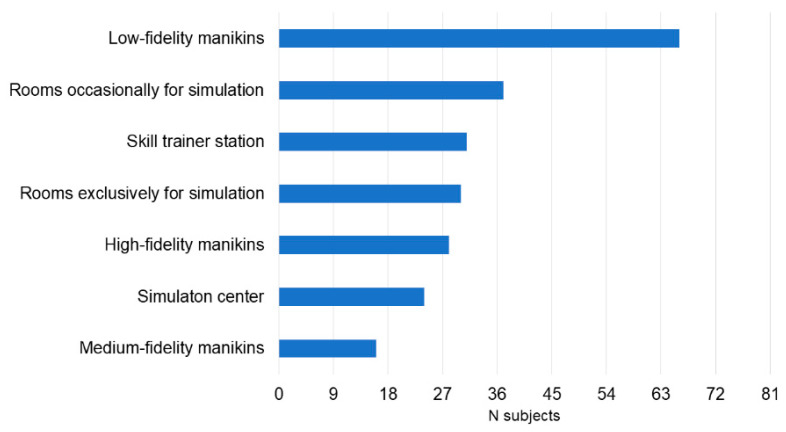
Simulation tools regularly available in the residency programs.

**Figure 3:**
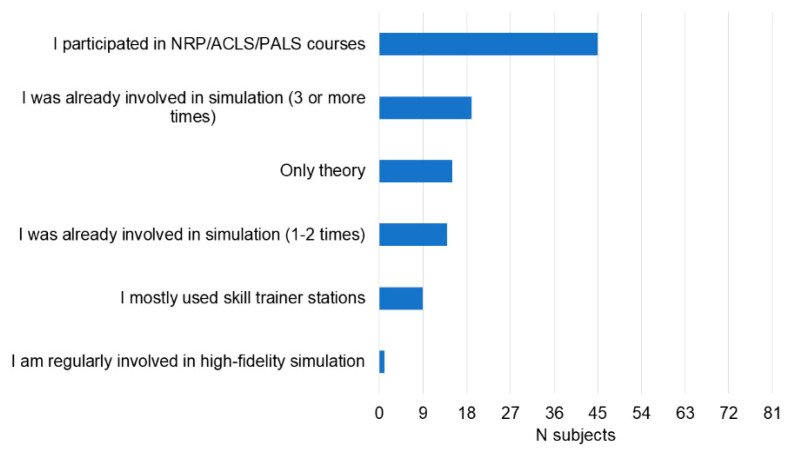
Knowledge on simulation before training for the event.

The participants trained for the event for one (*n* = 22, 27%), two (*n* = 35, 43%), three (*n* = 20, 25%), four (*n* = 2, 2%) or five (*n* = 2, 2%) months. Training before the event mostly included reviewing protocols and the reference textbook (86%) and low-fidelity simulations (70%). Most participants received educational support from the trainers in their residency program (77%) (Figure 1). Low-fidelity manikins were the most available simulation tools available in their residency programs (89%) (Figure 2). Before training for the event, knowledge on simulation was mainly based on their participation in standard resuscitation courses (56%), including the AHA Neonatal Resuscitation Program, Pediatric Advanced Life Support (PALS), and Advanced Cardiac Life Support (ACLS) courses. Even if 30% of participants had access to a simulation center during the training period, only one participant (1%) was regularly involved in high-fidelity simulation (Figure 3). 

### 3.4. Competition

**Figure 4:**
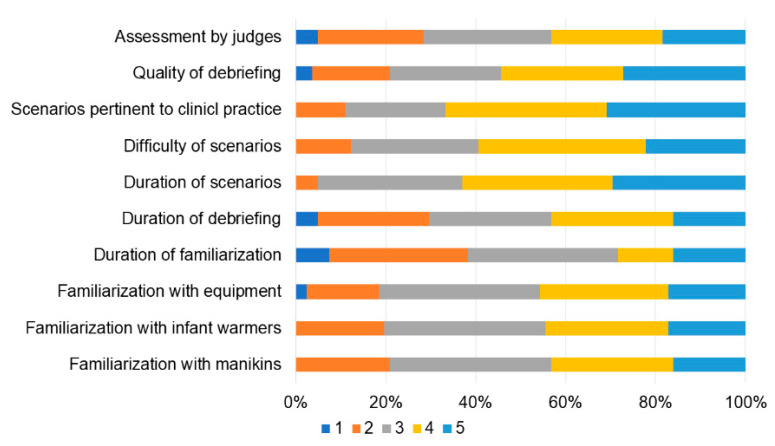
Participants’ satisfaction about aspects of the event (from 1 “unsatisfied” to 5 “very satisfied”).

**Figure 5:**
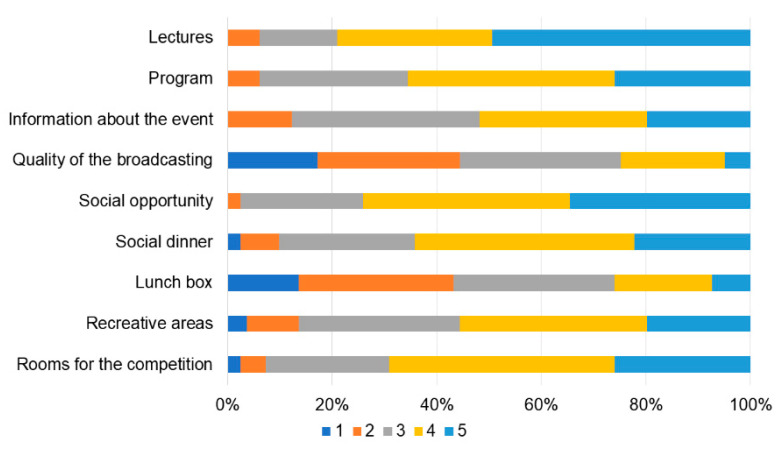
Participants’ satisfaction about organizational aspects (from 1 “unsatisfied” to 5 “very satisfied”).

**Figure 6:**
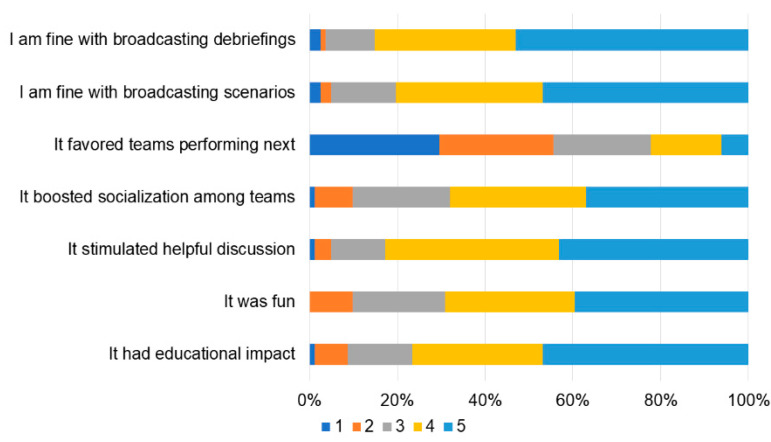
Participants’ satisfaction about the live broadcast of scenarios and debriefings in the auditorium (from 1 “totally disagree” to 5 “totally agree”).

**Figure 7:**
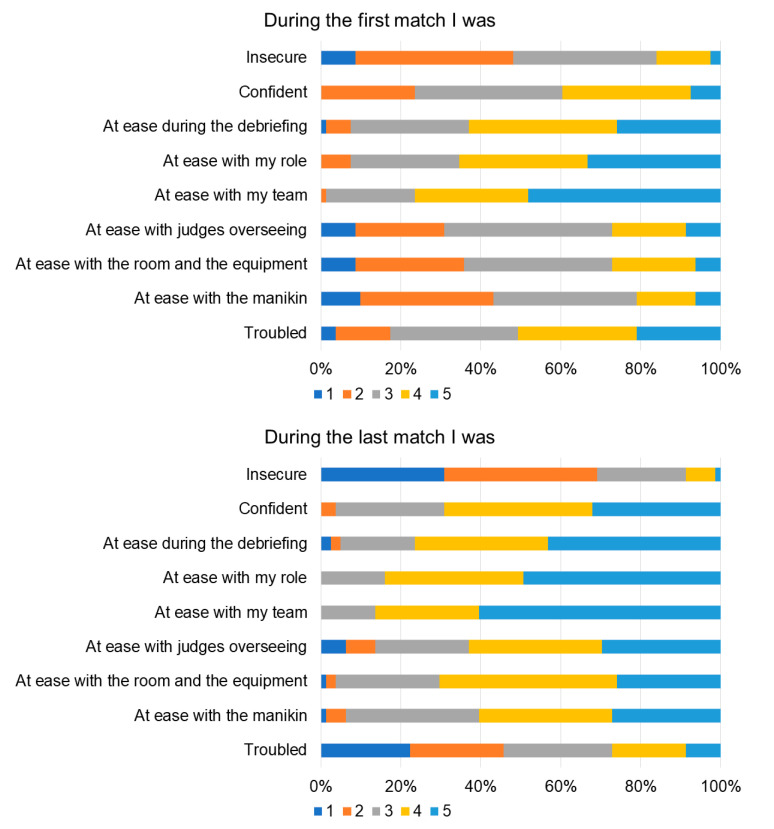
Participants’ confidence about aspects of the competition in the first match (**top**) and last match (**bottom**) (from 1 “not at all” to 5 “very”).

Overall, the participants were satisfied with the event (Figure 4) and the organizational aspects (Figure 5). They appreciated the live remote broadcast of scenarios and debriefings in the auditorium (Figure 6), although they suggested that the quality of the broadcasting could be improved (Figure 5). Some participants also indicated that the duration of the time allotted for familiarization with the equipment and the manikins should be prolonged (Figure 4). Participants’ confidence about components of the competition during the first and last matches is shown in Figure 7. All items improved from the first to the last match (*p* < 0.01).

### 3.5. Final Considerations

**Figure 8:**
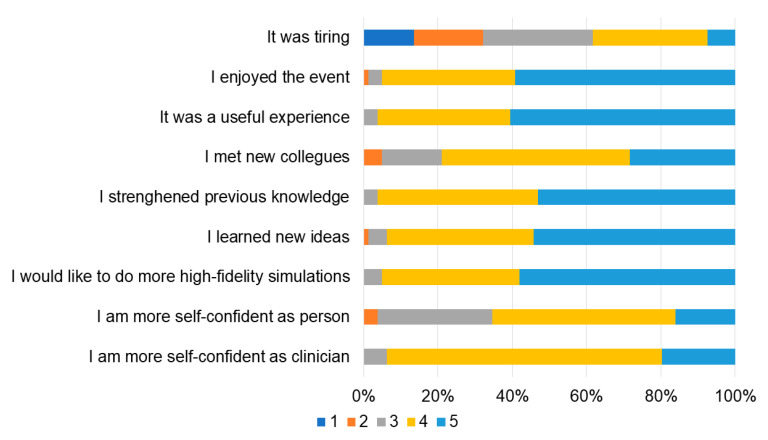
Take-home messages (from 1 “totally disagree” to 5 “totally agree”).

When the participants were asked to rate the event from 1 (very poor) to 10 (very good), the median rating was 9 (IQR 8–10), and 77 of them (95%) stated they would participate in another edition of this event. Overall, most participants felt that the event improved their knowledge and self-confidence related to neonatal resuscitation and stimulated them to become more involved in high-fidelity simulation (Figure 8).

## 4. Discussion

This report describes the implementation of a simulation competition on neonatal resuscitation for Italian pediatric residents and their perception of the educational experience. The event took inspiration from previously described events at simulation conferences, called SimWars, and used high-fidelity manikins representing a full-term neonate. To our knowledge, our event was the first simulation competition focused on neonatal resuscitation for pediatric residents. The level of participation from residency programs throughout the country was very high, and the residents had very positive perceptions of the educational impact of the event. They felt that participation in this simulation competition increased their knowledge and improved their self-confidence.

Overall, the participants were highly satisfied with the event and its organizational aspects. The most appreciated aspects were the pertinence, the duration, and the difficulty of the scenarios, confirming the appropriateness of the choice of the simulated cases. Of note, participant’s satisfaction can be a good indicator of an educational format, as it has been associated with increased learning motivation and involvement in the activities [19,20]. However, there was room for improvement regarding the time allotted for familiarization with the environment and manikins and the feedback shared during the debriefing and assessment by the faculty judges. Time spent becoming familiar with the simulation manikins, supplies, and equipment was particularly important during this event because we used several different high-fidelity manikins. Although the supplies and equipment provided were based on the reference textbook’s recommended equipment checklist, there were minor variations in the design of equipment and supplies between manufacturers and we found that some participants were not familiar with the brand provided. Based on these results, the organizing committee intends to dedicate more time on the first day of the competition in future editions to orientation and familiarize all participants with the simulation environment, manikins, supplies, and equipment. In addition, the organizing committee recognized a need to improve the structure and content of the debriefings provided by the faculty judges during the post-match debriefings. 

Our event included a live broadcast in the auditorium where the participants waiting for their next match could watch scenarios and debriefings of the ongoing match in simulation station #1. This was carefully planned to improve the educational impact of the event, as observational learning is an important learning method [21]. The residents overall appreciated the live broadcasts of scenarios, although the audio and visual quality of the broadcast could be improved. In addition, some felt that the live broadcast in the auditorium might provide an advantage to the teams performing in next competition, but this competitive advantage applied to any team rotating in the simulation stations. Importantly, the live broadcast increased the number of educational opportunities for the participants, thus contributing to the overall learning objectives of the competition and the cumulative learning of the participating residents, which was the main goal of the competition format. Of note, achieving proficiency in complex neonatal resuscitation situations has becoming increasingly difficult for Italian pediatrics residents because of the recent changes in the national residency programs (which tripled the number of the medical residents within the last 10 years) and the decreasing birth rate in the country, both contributing to a decrease in the number of learning opportunities for pediatric residents [22]. 

This relatively high intensity 3-day event exposed each participant to a minimum of five matches using realistic scenarios that they may experience in their clinical environment. While the competition day was the focus of the event and provided the motivation for teams to prepare, it was only a component of the larger educational design. Combined with the observational learning from the live remote broadcast, feedback from expert faculty, skills stations used for practice at the competition site between matches, self-directed learning with the reference textbook prior to the competition day, and team training that participants received from their local faculty while preparing for the event (up to 5-month duration), participants were exposed to multiple learning modalities over an extended time period. The take-home messages expressed by the audience overall reflected the educational impact of this format.

Literature suggests that participating in a competition may offer the chance to mature in both psychomotor and emotional domains of learning [11,12,23,24]. Competition is stressful, particularly when the skill being assessed is your ability to perform life-saving skills for a newborn and your performance is being judged by a panel of expert faculty. However, evidence suggest that the associated stress may be beneficial [25]. Our participants felt that their self-confidence about elements of the competition improved from the first to last match, and the event enhanced their knowledge and self-confidence overall. Self-confidence and the belief that you can be successful, self-efficacy, plays an important role in learning attitude and commitment to achieve goals and proficiency [26].

Beyond the participants’ satisfaction with the event, the survey provided insight about the knowledge and availability of simulation training in Italian pediatric residency programs. Our data suggested that the involvement of residents in simulation activities and the availability of high-fidelity manikins and dedicated areas for simulation should be improved; however, we acknowledge that the associated costs and human resources may be a barrier for implementation. This influenced the level of training for the event, which was mainly based on reviewing protocols, studying the suggested textbook, and participating primarily in low-fidelity simulation with local trainers. Given the decreased opportunities for Italian pediatric residents to gain real-life experience in high risk but low occurrence clinical scenarios in delivery rooms, it is even more important for residency programs to regularly schedule simulation training. 

In addition, the survey provided logistic information to improve the next planned edition of this simulation competition. Although we experienced nearly full participation from the Italian pediatric residency programs, there was limited logistical support for the residents. Few participants were offered educational leave to attend the competition despite the educational value of the event, and most resident participants had to cover their own travel and lodging costs. To ensure the ongoing success of future editions of this educational experience and similar experiences in other countries, the organizing committee believes that residency programs must be strongly encouraged to support participation by providing funds to cover travel expenses and assigning educational leave days. Providing this financial support is a tangible way for residency programs to demonstrate their commitment to resident education and the importance of simulation training.

Our survey has some limitations that should be acknowledged. First, the generalizability of the findings to other residency programs and other settings is unclear; however, we believe that our experience is useful for faculty planning a simulation competition for residents involved in other emergency situations. Moreover, the teams had heterogeneous post-graduate experience as might occur in real life situations. In this competition, most participants were pediatric residents in their 4th or 5th year of clinical training who should already have significant clinical exposure. In the post-event survey, we did not ask about the number of actual neonatal resuscitations each trainee had previously experienced, which would have provided some clinical basis for comparing participants’ perceptions of the educational experience. Second, the findings might be prone to participant selection bias because residents who chose not to participate in the event might have a different level of confidence in their resuscitation skills and a different level of interest in neonatal resuscitation. As a result, if participation had been mandatory for all pediatric residents, we might have obtained different results. We acknowledge that an unselected population of pediatric residents might differently benefit from the simulation competition event. Third, the participants self-assessed their confidence at two time points during the event (after the first and the last matches), but we did not include an objective evaluation of the improvement of their knowledge and technical and non-technical skills after participating in the event. Moreover, we were not able to assess the actual clinical performance of residents before and after the competition as the participants came from 31 residency programs from all across the country. We did not have a tool to assess either their perception or their actual performance in their clinical environment. Hence, we can only speculate that the positive self-perceived educational impact of the event may translate into improved clinical competence. Future studies should include assessments of performance in the actual clinical environment both before and after participation in the simulation event. Ideally, these assessments would be measured over several intervals to determine if measures of self-confidence, knowledge, clinical skills, and teamwork deteriorate over time and whether repeated exposures to simulation in the local environment mitigate this effect. 

Despite medical simulation having gathered great interest for learning purposes, it is still unclear that the evaluation of the effectiveness of this approach produces reliable or accurate results [27,28]. Although the simulation environment tries to replicate the stress of a real situation as much as possible, the participants are acting in a safe and controlled environment. In our event, the competition nature of the matches between two teams explicitly implied that they were being compared in terms of their performance during the simulation. Teams were aware they were being scored with both objective and subjective measures of team performance and clinical skills and a “winner” would be declared. The additional stress created by the competition nature of our event is likely different than the stress encountered in the clinical environment. Clearly, actual clinical care focuses solely on the outcome for the patient and does not involve competition between teams. Although we acknowledge that such evaluation may not translate into clinical competence, a competition requires a tool to identify the winner of a match. In our event, we considered the principles of reliability and validity of such a tool [28] by using a standardized evaluation form, a list of case scenarios that were prepared before the event by experts, and by having both teams of a match competing in the same scenario. Although our evaluation form was based on criteria described in the reference textbook, our faculty found the tool difficult to use in real-time during the simulation scenarios. For future editions of the competition, we plan to develop and validate a revised scoring tool that is more efficient and can be used in both a simulation setting and during actual clinical care.

Within its limitations, our experience may serve as an educational model for other institutions. Furthermore, the participants provided useful indications for improving the next edition of the event. First, the next edition may include an objective evaluation about knowledge and technical and non-technical skills before and after the competition to evaluate its impact as a learning experience. In addition, the evaluation of clinical performance before and after the competition by a local supervisor may provide information on the translation into clinical competence. However, this may introduce an evaluation bias as the local supervisor is familiar with the residents, but using external assessors would be very difficult due to logistics and organizational resources. Second, the next edition will collect more detailed information about participants’ experience before the event (such as the number of actual neonatal resuscitations each trainee had experienced) to provide some clinical basis for comparing participants’ perceptions of the educational experience. Third, organizational improvements will include more time for familiarization with manikins and equipment and improved communication between the judges and the participants during the debriefings (i.e., explaining the reasons underlying the scores). In addition, the audio and visual quality of the broadcast will be improved. Fourth, the evaluation form for the judges is currently under review by a panel of international experts using the Delphi method. Finally, the promotion of the event will be improved to encourage the residency programs to provide funds for expenses and days for educational leave for the event.

## 5. Conclusions

Participants appreciated the simulation competition on neonatal resuscitation for pediatric residents and were willing to participate in another edition. They self-perceived the educational impact of the event and felt that it improved their knowledge and self-confidence. Our findings suggest areas of improvements for further editions and may serve as an educational model for other institutions.

## Figures and Tables

**Table 1:** Summary of case scenarios used in the simulations.

Scenario	History	Examples of Possible Evolutions according to the Interventions by the Team
1	Post-term delivery of a newborn with meconium-stained amniotic fluid	Meconium aspiration, plugged endotracheal tube Pulmonary hypertension Unilateral or bilateral pneumothorax
2	Term delivery of a baby with cyanotic congenital heart disease in a rural hospital	Need for transfer to a tertiary hospital, using prostaglandin for maintaining ductal patency
3	Preterm caesarean delivery at 28 weeks’ gestation, premature rupture of the membranes, and Group B streptococci-positive vaginal swab	Unilateral or bilateral pneumothorax Pneumonia
4	Caesarean delivery of a late preterm with transient tachypnea	Stabilization with continuous positive airway pressure Unilateral or bilateral pneumothorax if exceeded with respiratory support
5	Operative vaginal delivery complicated with massive subgaleal hemorrhage	Advanced fluid/drug resuscitation
6	Bilateral hydrothorax in pregnancy complicated with Parvovirus B19 infection	Thoracic drainage placement
7	Congenital diaphragmatic hernia that was not diagnosed antenatally	Pulmonary hypertension Tension pneumothorax
8	Myelomeningocele	Placement of the newborn on latex-free foam/“doughnut” made with towels
9	Emergency cesarean section for fetal distress in a full-term pregnancy	Extensive resuscitation including chest compressions and medications
10	Emergency cesarean section in a pregnant woman with abundant vaginal bleeding (full-term pregnancy)	Extensive resuscitation including chest compressions and medications (and blood transfusion)

## Data Availability

The data presented in this study are available on request from the corresponding author.

## Supplementary Materials

The following supporting information can be downloaded at: https://www.mdpi.com/article/10.3390/children10101621/s1. Supplementary File S1: Evaluation form. Supplementary File S2: Online survey.

## References

[1] American Academy of Pediatrics (2021). Textbook of Neonatal Resuscitation (NRP).

[2] Madar J., Roehr C.C., Ainsworth S., Ersdal H., Morley C., Rüdiger M., Skåre C., Szczapa T., Te Pas A., Trevisanuto D. (2021). European Resuscitation Council Guidelines 2021: Newborn resuscitation and support of transition of infants at birth. Resuscitation.

[3] Halamek L.P., Cady R.A.H., Sterling M.R. (2019). Using briefing, simulation and debriefing to improve human and system performance. Semin. Perinatol..

[4] Bond W.F., Deitrick L.M., Arnold D.C., Kostenbader M., Barr G.C., Kimmel S.R., Worrilow C.C. (2004). Using simulation to instruct emergency medicine residents in cognitive forcing strategies. Acad. Med..

[5] Marshall S.D., Flanagan B. (2010). Simulation-based education for building clinical teams. J. Emerg. Trauma Shock..

[6] Stritzke A., Murthy P., Fiedrich E., Assaad M.A., Howlett A., Cheng A., Vickers D., Amin H. (2023). Advanced neonatal procedural skills: A simulation-based workshop: Impact and skill decay. BMC Med. Educ..

[7] Jamal M.H., Wong S., Whalen T.V. (2014). Effects of the reduction of surgical residents’ work hours and implications for surgical residency programs: A narrative review. BMC Med. Educ..

[8] Moonesinghe S.R., Lowery J., Shahi N., Millen A., Beard J.D. (2011). Impact of reduction in working hours for doctors in training on postgraduate medical education and patients’ outcomes: Systematic review. BMJ.

[9] Barsom E.Z., Graafland M., Schijven M.P. (2016). Systematic review on the effectiveness of augmented reality applications in medical training. Surg. Endosc..

[10] Nevin C.R., Westfall A.O., Rodriguez J.M., Dempsey D.M., Cherrington A., Roy B., Patel M., Willig J.H. (2014). Gamification as a tool for enhancing graduate medical education. Postgrad. Med. J..

[11] Ingrassia P.L., Franc J.M., Carenzo L. (2018). A novel simulation competition format as an effective instructional tool in post-graduate medical education. Adv. Simul..

[12] Weng Y.-H., Kuo K.N., Yang C.-Y., Liao H.-H., Chen C., Lo H.-L., Lee W.-C., Chiu Y.-W. (2013). Effectiveness of national evidence-based medicine competition in Taiwan. BMC Med. Educ..

[13] Kerfoot B.P., Kissane N. (2014). The use of gamification to boost residents’ engagement in simulation training. JAMA Surg..

[14] Okuda Y., Godwin S.A., Jacobson L., Wang E., Weingart S. (2014). SimWars. J. Emerg. Med..

[15] Dong C., Goswami R., Sim G.G., Kowitlawakul Y. (2015). Emergency medicine staff’s perception of SimWars: A Singapore view. Proc. Singap. Healthc..

[16] Dong C., Clapper T.C., Szyld D. (2013). A qualitative descriptive study of SimWars as a meaningful instructional tool. Int. J. Med. Educ..

[17] Cheng A., Kessler D., Mackinnon R., Chang T.P., Nadkarni V.M., Hunt E.A., Duval-Arnould J., Lin Y., Cook D.A., Pusic M. (2016). Reporting guidelines for health care simulation research: Extensions to the CONSORT and STROBE statements. Adv. Simul..

[18] R Core Team (2023). R: A Language and Environment for Statistical Computing.

[19] Baptista R.C.N., Martins J.C.A., Pereira M.F.C.R., Mazzo A. (2014). Students’ satisfaction with simulated clinical experiences: Validation of an assessment scale. Rev. Lat. Am. Enferm..

[20] Prion S. (2008). A practical framework for evaluating the impact of clinical simulation experiences in prelicensure nursing education. Clin. Simul. Nurs..

[21] van Gog T., Paas F., Marcus N., Ayres P., Sweller J. (2009). The mirror neuron system and observational learning: Implications for the effectiveness of dynamic visualizations. Educ. Psychol. Rev..

[22] Ministero della Salute. https://www.salute.gov.it/portale/news/p3_2_1_1_1.jsp?lingua=italiano&menu=notizie&p=dalministero&id=5553.

[23] Htwe T.T., Sabaridah I., Rajyaguru K.M., Mazidah A.M. (2012). Pathology crossword competition: An active and easy way of learning pathology in undergraduate medical education. Singap. Med. J..

[24] Cortez E.J., Boulger C.T., Eastin T., Adkins E.J., Granitto E., Pollard K., Bahner D.P. (2014). The ultrasound challenge 2.0: Introducing interinstitutional competition in medical student ultrasound education. J. Ultrasound Med..

[25] Demaria S., Bryson E.O., Mooney T.J., Silverstein J.H., Reich D.L., Bodian C., Levine A.I. (2010). Adding emotional stressors to training in simulated cardiopulmonary arrest enhances participant performance. Med. Educ..

[26] Surcouf J.W., Chauvin S.W., Ferry J., Yang T., Barkemeyer B. (2013). Enhancing residents’ neonatal resuscitation competency through unannounced simulation-based training. Med. Educ. Online.

[27] McInerney N., Nally D., Khan M.F., Heneghan H., Cahill R.A. (2022). Performance effects of simulation training for medical students—A systematic review. GMS J. Med. Educ..

[28] Bewley W.L., O’Neil H.F. (2013). Evaluation of medical simulations. Mil. Med..

