# Supplementary material for: A Simulation Competition on Neonatal Resuscitation as a New Educational Tool for Pediatric Residents
Source: Children (Basel). 2023 Sep 28;10(10):1621. doi: 10.3390/children10101621 (PMC10605553; doi:10.3390/children10101621)
Supplement: Supplementary file 1 [file children-10-01621-s001.zip › children-2574551-supplementary.pdf]

Supplementary File S1. Evaluation form.

NEONATAL RESUSCITATION COMPETITION
Padova February 16th-18th 2023

SCENARIOS' EVALUATION SHEET

**PREPARATION**	
**Thermal control**	
Delivery room temperature (optional)	
Radiant heater/infant warmer starting	
Warm dry towels	
Cap and polyethylene wrapping (if ≤32 weeks' gestation)	
**Suctioning**	
Suction source not exceeding 120 mmHg	
8 Fr catheter / large bore catheters (12-14 Fr) for meconium aspiration syndrome	
**Assessment and monitoring**	
Pulse-oximeter	
Stethoscope	
3-lead ECG	
**Ventilation devices**	
Set T-piece resuscitator	
Self-inflating bag (with pop-off valve check)	
Air/oxygen flows set up	
Appropriately sized facemask	
Laryngeal mask	
Endotracheal tubes	
Laryngoscope	
**Drugs (if indicated, eg. history of placental abruption)**	
Adrenaline 1 ml syringe (1:10.000 dilution)	
Volume expander syringe 40 ml (normal saline)	
Umbilical catheter	

**RESUSCITATION**	
**First steps**	
Timer starting	
Drying	
Wet towel removal	
Suctioning mouth-nasopharynx (if needed)	
Placing of uncovered baby under the infant warmer	
Polyethylene wrapping (if ≤32 weeks' gestation)	
Tactile stimulation (appropriate site and timing)	
Heart rate evaluation	
Saturation probe placement	
Correct site saturation probe	
**Positive pressure ventilation (PPV)/CPAP**	
PPV starting <1'	
Rapid evaluation of facemask PPV effectiveness	
Corrective maneuvers: "MrSOPA"	

Alternative airway: laryngeal mask	
Alternative airway: endotracheal tube	
Endotracheal tube: correct size	
Intubation attempt duration <30’’	
Correct placement: tip-to-lip distance	
Corrective maneuvers: “DOPPES”	
Appropriate CPAP application (eg. labored breathing / persistent cyanosis or desaturation)	
Appropriate choice of FiO2 according to SaO2 and minutes of life	
**Chest compressions**	
Start after 30’’ of effective PPV if heart rate still <60 bpm	
Alternative airway before starting compressions (if intubation failed, laryngeal mask accepted)	
Correct site for chest compressions	
Technique for chest compression	
Correct rhythm	
FiO2 1.00 associated with chest compressions	
Chest compressions timing before reassessment (1’)	
**Drugs active resuscitation**	
Start after 60’’ of effective PPV and chest compressions if heart rate still <60 bpm	
Adrenaline	
Correct site of administration (endotracheal/umbilical)	
Appropriate dosing (0.1-0.3 ml/kg IV; 0.5-1ml/kg ET)	
Bolus injection	
Following saline solution flush	
Correct timing (before volume expanders)	
Volume expanders (normal saline)	
Correct site of injection (umbilical)	
Appropriate dosing (10 ml/kg)	
Correct timing (5-10 min)	
If heart rate still <60 bpm: repeat cycle of drug active resuscitation	
**OTHER MEANINGFUL ACTIONS**	
Ask for arterial cord blood gas analysis just after birth	
Consider passive cooling at the end of resuscitation maneuvers where there is significant risk of moderate/severe hypoxic-ischemic encephalopathy	
Correct chest drainage if pneumothorax (site, devices)	
Time recording during resuscitation	

**NON TECHNICAL SKILLS**	
Briefing (role assignment/division)	
Order during preparation	
Team communication	
Leadership	
Reassessment	
Asking for help (eg. role exchange after failed intubation/ brainstorming during reassessment)	
Others	

**TOTAL SCORE**	

Each item gets one point. In the event of a tie, the winner will be decided during the final debriefing.

Supplementary File S2. Online survey.

Survey: Neonatal Resuscitation Competition 2023

Thank you for participating in the I Edition of the Neonatal Resuscitation Competition. After competing and being evaluated, now it's your turn to evaluate our work: this was the first edition of this event, and we would like to understand how we performed. The answers are anonymous and are presented in an aggregate modality.
By replying, you consent to the use of data for statistical and research purposes.

Section 1: Tell us who you are.

1. Where do you come from? Your University / School.
2. Year of specialization?
    a. 0
    b. 1
    c. 2
    d. 3
    e. 4
    f. 5 (Select 0 if you are not a student)
3. In the competition you arrived at:
    a. Groups
    b. Eighths
    c. Quarters
    d. Semifinal - Final
    e. I was in the faculty
    f. I did not play

Section 2: Your journey. Tell us something about your travel to Padua.

4. How many were you on your team? Include everyone who came to Padua, including reserves, coaches, any members, ...
    a. 1
    b. 2
    c. 3
    d. 4
    e. 5
    f. 6
    g. 7
    h. 8
5. How far did you travel to come to Padua?
    a. < 200km
    b. 200 - 400km
    c. 400 - 600km
    d. 600 - 800km
    e. > 800km
6. Means of travel?
    a. Airplane
    b. Train
    c. Car sharing (e.g. BlaBla car or similar)

    d. Bus (e.g. Flixbus)
    e. Personal car
7. How did you organize with your daily work?
    a. I had to take time off
    b. I asked not to work those days and to recover later
    c. I was removed from shifts specifically for this event
    d. Other…
8. Expenses: Mark which of these expenses were your personal responsibility, and which instead have been/will be reimbursed.

	At my expense	Refund
Accommodation		
Food		
Travel		

9. How much was the total cost approximately and how much did you pay?

	< 200eu	200-400 eu	400-600 eu	600-800 eu	> 800 eu
Total cost					
Costs at your expence					

10. Do you think a dedicated grant may be useful to support the participants for this event?
    a. Absolutely not
    b. No I do not think so
    c. I don't know
    d. I think so
    e. Absolutely yes

Section 3: The preparation. Tell us how you prepared for the competition.

11. How many months have you been preparing for the competition?
    a. 1
    b. 2
    c. 3
    d. 4
    e. 5 (mark 5 to indicate 5 months or more)
12. How did you prepare for the competition? (multiple answers allowed)
    a. I did some workouts with my mates
    b. I was able to train regularly with the team
    c. I had the support of instructors from my school
    d. I had the support of instructors outside my school
    e. I trained on technical skills
    f. I trained with low fidelity simulations
    g. I trained with high fidelity simulations
    h. I read the protocols/manual of neonatal resuwscitation
13. Which simulation tools do you normally have access to?

	No	Yes	Yes, in the School / Department	Yes, in the University	Yes, in the Hospital
Simulation Center					
Skill trainers					
Low Fidelity Mannequins					

Medium fidelity dummies					
High fidelity mannequins					
Rooms used for simulation					
Convertible rooms for simulation					

14. How familiar were you with simulation prior to this event? Training made specifically for the competition should be excluded.
    a. None
    b. I had mainly used skill trainers
    c. I participated in neonatal resuscitation/ACLS/PALS training
    d. I had already simulated 1-2 times
    e. I had already simulated several times
    f. I regularly do high fidelity simulations

Section 4: Tell us how you felt during the competition and what you think about the event.

15. Satisfaction. Evaluate your satisfaction on the following aspects with a score from 1 (not at all satisfied) to 5 (very satisfied)
    a. Explanations on manikins
    b. Explanations on the infant warmers
    c. Explanations on principals
    d. Duration of familiarization
    e. Duration of debriefings
    f. Duration of scenarios
    g. Difficulty of scenarios
    h. Relevance of the scenarios to the clinical activity
    i. Quality of the debriefing
    j. Judges' ratings
16. During the competition, scenarios and debriefings were broadcast live in the auditorium. Tell us what you think about it. Rate your opinion from 1 (strongly disagree) to 5 (strongly agree)
    a. It was formative
    b. It was fun
    c. It was a conversation starter
    d. It helped to socialize between teams
    e. It favored who played later
    f. I am in favor of broadcasting the scenarios
    g. I am in favor of broadcasting debriefings
17. During my first scenario, I felt… Rate the following statements from 1 (not at all) to 5 (very much)
    a. Insecure
    b. Confident
    c. At ease during the debriefing
    d. At ease with my role
    e. At easy with my team
    f. At ease with judges overseeing
    g. At ease with the room and the equipment
    h. At ease with the manikin
    i. Troubled

18. During my last scenario, I felt… Rate the following statements from 1 (not at all) to 5 (very much)
    a. Insecure
    b. Confident
    c. At ease during the debriefing
    d. At ease with my role
    e. At easy with my team
    f. At ease with judges overseeing
    g. At ease with the room and the equipment
    h. At ease with the manikin
    i. Troubled
19. The event in general: evaluate the following aspects of the organization with a mark from 1 (very poor) to 5 (very good).
    a. Competition rooms
    b. Spaces and areas used out of competition
    c. Launch box
    d. Social dinner
    e. Possibility to socialize
    f. Broadcast quality in the lecture hall
    g. Logistics information (e.g. event venue)
    h. Plan
    i. Master classes
20. Is there something you expected but was missing / could be improved?
21. Is there something you didn't expect but there was?
22. What do you take home? Please rate your opinion on these statements from 1 (strongly disagree) to 5 (strongly agree).
    a. I feel more confident about myself as a doctor
    b. I feel more confident in myself as a person
    c. I want to do more high fidelity simulations
    d. I learned new ideas
    e. I reinforced notions I knew
    f. I expanded my network of contacts
    g. It was a useful experience
    h. It was fun
    i. It was tiring
23. Overall, how would you rate this event?
    a. 1
    b. 2
    c. 3
    d. 4
    e. 5
    f. 6
    g. 7
    h. 8
    i. 9
    j. 10
24. Would you like to participate again if it were to be done again next year?
    a. Sure
    b. Perhaps

c. No, I do not think so

25. Write us any notes, advice, suggestions, or complaints.
